# Risk and Blameworthiness by Degree

**DOI:** 10.1007/s10790-021-09798-x

**Published:** 2021-02-18

**Authors:** Adriana Placani, Stearns Broadhead

**Affiliations:** Institute of Philosophy, University of Graz, Graz, Austria

## Introduction

It seems intuitively plausible to hold the view that, *prima facie*, one is more blameworthy for imposing a greater rather than a smaller risk. Other things being equal, it seems that an agent is more deserving of blame when they threaten you with a gun than when they threaten you with a pillow; an agent is more blameworthy when they drive their car without functioning indicators than when they drive their fully functioning car; an agent is more blameworthy when emitting more rather than fewer toxins in the air.^1^ Moreover, the legal realm functions in accordance with much of the same insight through the incorporation of a principle of proportionality in punishment. Perpetrators are punished, in part, proportionally with the amount of harm or risk they create. Thus, it seems that both common sense morality and the law dictate that, at least *prima facie*, degrees of blameworthiness ought to track degrees of risk impositions in the sense that, the higher the risk imposed, the greater the deservedness of blame. Hereafter, this line of reasoning is dubbed the ‘blameworthiness tracks risk thesis’ (BTRT).

The BTRT seems incontrovertible, almost banal. However, BTRT is under strain from, at least, two problems: the reference class problem and the mental state of the agent problem. The first is a risk-specific matter that distinguishes itself through its implication that no risk determination is unequivocal. It follows, then, that no blame attribution on the basis of risk imposition would be unequivocal. Thus, the reference class problem undermines the BTRT.

The second problem, the mental state of the agent, also challenges the plausibility of the BTRT. This is because when harm is intended by means of risking that harm (i.e., intentional harming), then whether the risk through which the harm is pursued is greater or lesser can make no moral difference to the deservedness of blame of that agent. In other words, an agent can be blameworthy to the same degree for creating a 10% or 90% chance that someone will lose their eyesight, when they intend that loss.

This essay explores these two challenges and probes the strength of the BTRT in light of them. Further, it argues that these problems and their attendant complications undermine the BTRT even if some avenues for its preservation exist. Before exploring these matters, a few preliminaries are in order. Section one provides a brief survey of core concepts and conceptions associated with moral responsibility, blameworthiness, risk, as well as the BTRT itself. Additionally, the section addresses the appropriateness of using a notion such as degrees when it comes to blameworthiness and risks. The second section of this work illustrates the reference class problem and the ways in which this poses a threat to the BTRT. Moreover, the section sketches out a way for the BTRT to avoid the reference class problem. In similar fashion, the third section accounts for the mental state problem in relation to the BTRT. It provides a succinct overview of how and why the problem arises, as well as a possible answer to it. The fourth section concludes this work.

## The Concepts: BTRT, Responsibility, Blameworthiness, Risk, and Degrees

The plausibility of the BTRT is hard to shake. Regardless of one’s conception of risk and its intrinsic moral significance (or lack thereof)^2^, the claim that the higher the risk an agent culpably creates for others, the higher her degree of blameworthiness, appears to jibe perfectly with both common intuition and the legal realm.

What lies at the heart of the BTRT is the purported correlation between degrees of blameworthiness and degrees of risk, but it might not be clear whether such talk of degrees is appropriate in the first place. Given this possible contention, the following addresses whether blameworthiness and risks admit of scalarity and provides a sketch of the main concepts and conceptions at work. Addressing these aspects of the BTRT helps give content to the thesis, which will be in focus at the end of the section.

In terms of its everyday understanding it seems perfectly straightforward that blame and blameworthiness come in degrees. We often use comparative adjectives to describe various feeling of resentment, indignation, blame, and so on, which we direct at others. We can say without much compunction that we are less resentful of an agent for her unjustifiable tardiness on a care-free day than we are for her tardiness on a day when the solving of an urgent matter required punctuality. In other words, our reactions to moral transgressions vary.

Talk of the emotions that are elicited by wrongdoings fits well with a Strawsonian view of responsibility. Roughly stated, Peter F. Strawson conceptualizes responsibility as an interpersonal relation in the sense that being responsible is fundamentally connected to the practice and norm of holding others to be responsible.^3^ In turn, holding responsible is understood in terms of morally reactive attitudes, which are emotional responses to other people’s actions. These reactions can vary and range wildly: from resentment, blame, contempt, outrage, and anger, all the way to gratitude and love.^4^ The following relies upon a broadly Strawsonian view and assumes that blameworthiness consists in the appropriateness of certain blame-related responses.^5^

In everyday life it is common to conceive of blame and blameworthiness as admitting of degrees (e.g., depending on the gravity of the outcomes, circumstances surrounding the agent or her actions). However, when an agent is said to be more or less blameworthy for something, there are a few things that could be meant: (1) the agent is deserving of more or less blame; (2) the agent is more or less deserving of blame; or, (3) a combination of the first two.

Regarding (1), if an agent is to be *justifiably* attributed with a greater or lesser amount of blame,^6^ then this attribution would need to follow, at least partly, from (2) the agent’s deservedness of that amount of blame (whatever it turns out to be).^7^ Thus, whatever degree of blame is to be attributed stands in need of justification, which seems to make (3) the more accurate view. Having said this, while it is common for people to exhibit variation in reactions, such as blame, it is, perhaps, less clear whether such variation exist with regards to deservedness itself.

However, there are reasons to regard the notion of deservedness, which is here used interchangeably with desert, as scalar. At least colloquially, someone can be said to be more or less deserving of something (e.g., promotion, reward, punishment) when the reasons on the basis of which she ought to receive that something are, themselves, stronger or weaker. Thus, we would probably not regard John to be as deserving of a promotion as Paul if Paul had greater experience and skills than John (even though John was also deserving of the promotion). It is not unusual for ranking to occur in such matters nor does it seem particularly problematic.

This kind of talk captures the fact that, at least in practice, blame and blameworthiness admit of degrees and are used as such (e.g., “you are more blameworthy than your sister,” “you deserve more blame”). But, perhaps, practice is leading us astray. So, the question becomes why should we understand blame and blameworthiness as scalar—that is, as admitting of degree?

Fundamentally, acknowledging the scalarity of blame and blameworthiness is a matter of fairness. It is a concern for fairness that makes speaking of degrees of blame and blameworthiness appropriate. The primary way in which we determine an agent’s (i.e., the subject of responsibility) deservedness of blame is by examining her relation to the object for which she is to be held responsible (e.g., harm). Relationships between objects and subjects of responsibility can vary widely (e.g., the object may have been intended by the subject, the object may have been unforeseeable to the subject).^8^ This variability should—out of fairness considerations—affect one’s degree of deservedness of blame. This suggests, then, that the scalar and varying nature of relationships between subjects and objects of responsibility, should translate into degrees of blameworthiness. Furthermore, awareness of and sensitivity to different relationships between subjects and objects of responsibility should not only affect one’s degree of blameworthiness, but also entail a suitable calibration of blaming responses.

Degrees of blameworthiness, then, have to do with how blameworthy an agent is for some action or outcome, which is a determination that is sensitive to the relationship that the agent bears towards that the object of responsibility assessment (e.g., the causal relationship between subject and object, the mental state of the subject vis-à-vis the object). Notwithstanding the foregoing, a full argument for the scalar nature of blameworthiness cannot be offered here; nevertheless, the following will operate under the assumption that talk of degrees in relation to blameworthiness is appropriate.^9^

Having provided some reasons why talk of degrees of blame and blameworthiness is fitting, it is time to probe this concern in relation to risks. The case for conceiving of risks in scalar fashion seems straightforward. The standard definition of risk, which is prevalent in philosophy and across a host of other disciplines, conceives of risks as admitting of at least two aspects: probability and severity.^10^ Both of these notions admit of gradation. The probability of an event, E, occurring can be greater or lesser, anything between 0 and 1. The severity of a harm occurring is an evaluative consideration that admits of ranges. In light of this, we will assume a scalar conceptualization of risk and take it to be uncontroversial. However, we will examine the strength of the BTRT by varying only the element of probability in relation to blameworthiness. In other words, when speaking about greater, lesser, higher or lower risks, what will be referred to is a greater, lesser, higher or lower probability of harm. Probability is taken as the varying element because of the ease with which the concept admits of gradations. Furthermore, varying severities of harm could needlessly complicate matters because of possible disagreements regarding the nature and/or quality of harms. Comparatively, probabilities are a far more straightforward matter in terms of their amenability to variance.

In spite of the unproblematic degree-related aspects of risks above, there are many contentious matters surrounding risk as a concept. Famously, risk has been marked by a rift between two opposing camps: an objective one that understands risks to be determined by facts in the physical world, and a subjective one that views them as social and cultural constructions.^11^ Objective accounts of risks typically rely on frequentist interpretations of probability.^12^ Frequentists view a risk of an event, E, as the frequency with which E occurs in the general population or some other reference class that is selected. The frequency with which the risk manifests in the reference class is taken to be an objective and scientifically verifiable fact. One difficulty with this view—referred to as ‘the reference class problem’^13^—is that the probability of an event can change depending on how it is classified, and the same event can be classified in a variety of ways on the basis of it belonging in different reference classes.

The subjective camp is very diverse, but one of the most influential accounts is the personalist or Bayesian conception.^14^ Broadly, this view considers a judgment of probability on the basis of particular individuals’ actual degree of belief, credence or confidence in a given proposition, which is typically measured on the basis of agents’ betting behaviors.^15^ Some versions posit that objective probabilities (e.g., frequencies, propensities) do not exist and that the only measure of probability is individualistic. One difficulty with this view is that probability judgments can vary broadly from person to person, especially in the absence of constraints on what ought to count as a rational belief.

Both the objective and subjective perspective on risks will be featured again. Although more can be said about these matters, the above aimed merely to introduce the main characteristics of these two prominent views. This brings us to the final piece of the conceptual puzzle: the blameworthiness tracks risk thesis itself and its plausibility.

It seems plain to say that, other things being equal, an agent is more blameworthy when she culpably (e.g., knowingly) creates an 80% chance rather than a 20% chance of some bad outcome. If the agent is certain that her actions will lead to the bad outcome, then her blameworthiness reaches its peak. It is almost beyond argument that our intuitions point to the conclusion that how much risk one, unjustifiably, imposes onto others is, at least *prima facie*, directly correlated with her blameworthiness. Still, the following two illustrations of BTRT in both the *ex post* (i.e., after the risk materializes into harm) and *ex ante* (i.e., before the risk materializes into harm) perspectives aim to show more of its appeal.

Consider the BTRT in the *ex post* variant and two versions of the same event. Suppose that in version (1) an agent, Rob, knowingly and unjustifiably unleashes an 80% risk of injuring Anne. *Ceteris paribus*, in version (2) Rob knowingly and unjustifiably unleashes a 20% chance of injuring Anne. Anne gets injured in both (1) and (2) and suffers the same exact injury. The difference is that Rob is more causally connected to Anne’s injury in (1) than in (2). This means that Anne’s injury is more straightforwardly attributed to Rob in (1) than in (2) rather than it being attributed to other factors (e.g., chance). This, in turn, would compel us to increase Rob’s blameworthiness in (1) than in (2).

Now, consider BTRT *ex ante* and adopt a consequentialist model. On some consequentialist version, Rob ought to consider the expected (dis)utility of his actions before deciding how to act. Let us consider again two versions of the same event: in version (1) Rob has a pistol with 10 chambers and puts a bullet in one of the chambers; in version (2), c*eteris paribus*, Rob has a pistol with 10 chambers and puts 5 bullets in the chamber. Rob calculates the expected disutility of firing his pistol at Anne and assigns a disutility of -100 to Anne’s dying. In (1), the expected harm is: -100 x 1/10 = -10; in (2), the expected harm is -100 x 5/10 = -50. Given that (2) carries more expected harm that (1), then embarking on (2) is harder to justify. Assuming neither pursuing (1) nor (2) is justifiable, then this would compel us to assign more blameworthiness in (2) than (1).

Moreover, the legal realm appears to also embrace the BTRT. Consider Andrew Ashworth’s discussion of recklessness where he writes: “recklessness . . . is usually defined in terms of awareness of risk, but the degree of culpability surely varies according to the magnitude of the risk.”^16^ Moreover, consider that foresight of virtual certainty or high degree of probability of consequences (i.e., the legal offense) can confirm the evidential presumption of intention whilst a lower probability can refute it. Intention carries the highest degree of culpability and is considered a more serious mental state than recklessness or negligence.

Finally, consider Andrew von Hirsch and Nils Jareborg who write that the “seriousness of a crime has two dimensions: harm and culpability. Harm refers to the injury done or *risked* [our italics] by the act; culpability to the factors of intent, motive and circumstances that determine the extent to which the offender should be held accountable for the act.^17^ In a similar vein, Von Hirsh and Richard Frase respectively posit that an agent’s degree of blameworthiness is determined by two elements: (a) the nature and seriousness of the harm caused or *threatened* [italics added] by the agent’s action and (b) the agent’s degree of culpability in performing the action. The second element, (b), depends on a multitude of factors, such as an agent’s mental capacity, mental state, and motives, while the first, (a), focuses on the risk of harm or harm produced by the agent.^18^

Assuming that the two elements of harm or risk of harm and culpability are vital for blameworthiness attributions, it is important to note that in further analysis of the BTRT the culpability element (e.g., mental state) is held constant. The element of blameworthiness that is varied is the risk in its probability aspect. This is because in order to determine whether the degree of risk matters directly to blameworthiness, the aspect of risk must be isolated from other factors that might influence the verdict of blameworthiness.

The intuitive appeal of the BTRT should be evident, but the above illustrated some of its force without exhausting its instantiations. Furthermore, and more generally, the section aimed to provide an overview of the main concepts that will be in operation throughout the rest of this work, as well as offer reasons why the quest and purpose of this work’s inquiry is appropriately constructed.

## BTRT and the Reference Class Problem

The following aims to illustrate the ways in which the BTRT can be undermined by the reference class problem, which was previously described. Moreover, the section will indicate one possible source of redress for the BTRT. Recall that the BTRT claims that there ought to be a direct correspondence between degrees of risk imposed and blameworthiness.

The objection stemming from the reference class problem takes the following form: Degrees of risk imposition ought not and cannot track blameworthiness because the probability of harm created varies with the reference class selected. The reference class problem arises because the probability of an event occurring can change depending on how it is classified, and the same event can be classified in a variety of ways on the basis of it belonging in different reference classes. Given that any determination of degrees of risk imposition must account for its probability feature (i.e., the probability that the risk will eventuate), the reference class problem applies to risk determination.

The reference class problem appears in John Venn, where he writes that: “It is obvious that every individual thing or event has an indefinite number of properties or attributes observable in it and might therefore be considered as belonging to an indefinite number of different classes of things.”^19^ To exemplify, we can consider attempting to assign a probability to the event that Tonya Harding will be dead by the age of 60. Tonya belongs to many different reference classes on the basis of which we can arrive at different probabilities: she is an American citizen, a woman, a former ice-skater, and a smoker. Which reference class is the one relevant for determining the probability of her death?

There is great ambiguity surrounding the identification of the appropriate reference class to which she belongs and on the basis of which we can approximate her chances of dying. Hans Reichenbach captures this problem well and dubs it accordingly: “If we are asked to find the probability holding for an individual future event, we must first incorporate the case in a suitable reference class. An individual thing or event may be incorporated in many reference classes, from which different probabilities will result. This ambiguity has been called the problem of the reference class.”^20^

In order to solve this issue, both Reichenback and A.J. Ayer proposed using the narrowest reference class for which reliable statistics can be compiled. 21 Indeed, this seems to be a sensible recommendation as the narrowest class would be the most faithful description (for which reliable frequencies exist) of the object of risk (e.g., Tonya Harding). However, there are some serious problems with this suggestion.

First, the notion of reliability in statistics can be easily challenged (e.g., on grounds of vagueness, bias).^22^ Second, there may be multiple classes that are equally narrow for which we have reliable statistics.^23^ This means that there may not be a non-arbitrary or, at least, no unambiguous way of choosing between equally narrow reference classes. Thirdly, even if we can identify the narrowest reference class, such a class would have been selected through a process of whittling down a wider reference class. However, the particular (wider) reference class chosen at the outset would itself be plagued by arbitrariness or ambiguity, as would the subsequent moves of delimiting subsets of this class.^24^

Finally, even if a single narrowest reference class existed, John Maynard Keynes points out that relying on such a class may make one blind to other relevant factors that are known, but for which there is no statistical or dependable evidence.^25^ Such factors could provide solid reasons for probability adjustments, but they are barred from consideration. In our example, if we knew that Harding was a car-surfing enthusiast, then such information would make it sensible to adjust higher the probability of her dying by the age of 60, but the constraints of statistical evidence would not allow it.^26^

Generally speaking, then, the worry is that any assignation of risk is at some level indefinite because there is no unequivocal way of assigning probabilities. 27 Risks that agents create for others are seemingly indeterminable in terms of probabilities or, in other words, with regard to the degree of risk created. This indeterminacy of risks can also make establishing blameworthiness based on the level of risk imposed by agents indeterminable. It follows that, inasmuch as the reference class problem exists for probability theories, the BTRT is undermined.

Consider a hypothetical that can capture the difficulty of the problem. John threatens Mary with a knife. One way to determine the amount of risk of harm that John unleashes onto Mary is by asking about the reference class into which Mary fits. Let’s say that Mary is a 34-year-old hemophiliac woman trained in jujitsu. Now, it would be absurd to hold concomitantly the following views: John is most to blame when Mary’s reference class is that of a hemophiliac, because the risk of harming a hemophiliac when holding a knife to her throat is very great; John is less to blame when Mary’s reference class is of a 34 year old female, because the risk of harming a 34 year old woman when holding a knife to their throat is lower as compared to a hemophiliac; and, John is the least to blame when Mary’s reference class is that of jujitsu masters, because the risk of harming a jujitsu master when holding a knife to her throat is lower as compared to the other reference classes.^28^ Further, we could easily devise yet other classes, which would only make matters worse for the BTRT. Of course, the narrowest reference class that Mary belongs to is of one—herself—but that is hardly something for which reliable statistical evidence can be said to exist.

Although there is no solution to the reference class problem with the resources of frequentist accounts, the subjective Bayesian account of probability, which was mentioned before, can provide an answer.^29^ According to the subjective theory: “Probability is . . . defined as the degree of belief of a particular individual, so that we should really not speak of the probability, but rather Ms. A’s probability, Mr. B’s probability or Master C’s probability.”^30^ Thus, in accordance with this account, in order to determine the probability of harm that John creates we ought to look at the degree of belief that he holds with respect to his chance of harming Mary.

So, what is the probability of harm that John’s action gives rise to? Objectively, this may be an unanswerable question. Subjectively, however, we can assess the amount of risk that John takes himself to be creating by performing the action. Needless to say, how much risk John believes himself to be creating matters for establishing his degree of blameworthiness. Such an approach makes blameworthiness for risk impositions into an entirely subjective matter. This strategy can rescue the BTRT because, when conceived in subjective terms, the blameworthiness of an agent ought to track the degree of risk that the agent believes herself to be imposing. So, if an agent takes herself to be imposing a lower risk of harm than a higher risk, then her degree of blameworthiness will be lessened.

This escape for the BTRT is in line with common thought on blameworthiness as it zeroes in on those conditions that matter for its assignation. Among such conditions, control features prominently.^31^ It is widely acknowledged that control is necessary for responsibility although what sort of control is needed is up for debate.^32^ It should be borne in mind that the issue of control is complex, and the following will eschew much of this complexity, in particular the free will debate. If control is taken to be a condition of responsibility, then we ought to inquire after those things that are within the control of the agent when establishing blameworthiness. What is under the control of our agent, John?

Although one cannot control how much risk they are in fact (i.e., objectively) imposing, they can control the amount of risk they decide to impose based on their own judgment. In our case, John has no control over Mary’s belonging to different reference classes, but, by hypothesis, he has control over his decisional and actional pursuit of harm. The best candidates, then, for criteria of blameworthiness that satisfy the control requirement seem to be John’s decision to and performance of the action of holding a knife to Mary’s throat. John decides to pursue a course of action that he believes carries a certain risk, and it is on this basis that his blameworthiness should be assessed.

It should be noted that we need not rely exclusively on an agent’s subjective beliefs in order to assess her culpability. We can also evaluate the reasons that she has for holding her beliefs and probe as to whether her beliefs are justified. In this way, we could also assess an agent’s degree of blameworthiness for holding certain beliefs. Holding mistaken beliefs may be blameworthy when the agent ought to have known better, ought to have taken steps towards acquiring requisite knowledge, or when the correct information was widely available (e.g., evidence about climate change).

We can assess the justifiability of having a certain belief regarding the risk that one decides to impose by determining the risk that a reasonable person would take herself to be creating by performing a certain action. Such a judgment would be relative to the available evidence and given rationality constraints. To return to the example, then, we could evaluate the amount of risk a reasonable person would assume that she was creating when deciding to act and acting in the exact ways in which John did and relative to the available evidence. If a reasonable person could not have known of Mary’s hemophilia under the circumstances, then we ought to strike this factor from the assessment of John’s blameworthiness.

Thus, it seems then that the reference class problem can be avoided when what are considered are subjective probabilities. However, although usually regarded to affect frequentist interpretations of probability in particular, some theorists have argued that certain versions of the subjectivist interpretation of probability are also vulnerable to the reference class problem.^33^ To be sure, if agents’ subjective probabilities are based or rely upon objective probabilities’ estimates (e.g., by utilizing frequencies), then it seems that the reference problem would, once again, rear its ugly head. Moreover, the above solution for the BTRT will be partially challenged in the next section, which illustrates that there is a problem in relying on the decision of agents to establish varying degrees of blameworthiness.

To sum up, this section illustrated the way in which the reference class problem undermines the BTRT when the conception of risk in operation focuses on objective probabilities. Moreover, the above suggested one avenue for the preservation of the BTRT by substituting the objective conceptualization of probability with a subjective one. The latter is not an unproblematic answer, but it is one that holds the potential for defending the BTRT against the reference class problem. This solution will be partially undermined in the next section.

## BTRT and the Mental State Problem

Having presented the first objection to the BTRT, we now arrive at the second identified problem that undermines the plausibility of the BTRT: the mental state of the agent. The problem of the mental state eventuates in some cases where harms are intentionally sought by means of risking. In certain instances of intentional risking, agents make a conscious decision to harm and act on the basis of this decision. A problem appears because, in such cases, the fact that agents unleash greater or lesser amounts of risk (through which they pursue the harm) can make no moral difference to their deservedness of blame.

To exemplify, suppose that Devin decides to blind his husband, Angel. Devin goes on the Darknet and finds a dangerous chemical, X, to add to Angel’s contact lens solution. In scenario A, the chemical that Devin finds available is not terribly potent: it offers Devin a 10% probability that Angel will go blind (set aside, for now, the theory of probability that this estimate attaches to). Devin purchases and uses chemical X. In scenario B, with the same degree of effort, Devin finds available a much more dangerous chemical, XX, that increases the probability of vision loss to 60%. Devin purchases and uses chemical XX.^34^ Is Devin more blameworthy in the second case as compared to the first? Recall that in both cases Devin wishes that his husband goes blind, makes the decision to bring this about, and, in both cases, he spends the same amount of effort to act in accordance with his decision (e.g., time, cost, etc. or, in other words, all else is equal).

The fact that chemical XX is available for purchase lies outside Devin’s control by hypothesis. Presumably, if XX had been available in scenario A, then Devin would have purchased and used it. This would indicate that Devin’s level of blameworthiness is, plausibly, equal in both cases.^35^ Why is this so? One way of making sense of this intuitive response is to focus, once again, on those things that are under Devin’s control.

In both scenarios, Devin harbors the same desire and forms the same decision (i.e., to pursue his desire).^36^ The difference between the two cases is that he ends up performing different actions. But, Devin’s performance of the respective action is constrained by factors that are outside of his control. This is not to say that the way in which Devin (or anyone) chooses to implement his decision is unimportant; it is, especially, if their choice is free. However, in the two scenarios Devin does not possess control over the availability of products, and so, he ends up performing different actions carrying different degrees of risk. Devin is not free with respect to how he implements his decision, but he is, by hypothesis, free to form the decision to blind Angel (i.e., he possesses decisional control). This decision, which is the same in both versions of the events, grounds his equal degree of blameworthiness.

When free, decisions are the instantiation of agents’ autonomy.^37^ Following Joseph Raz’s account (to a certain extent), fully-fledged decisions are taken to be ‘acts of the will’ that fulfill four conditions: (a) they embody an intention; (b) they are reached as a result of deliberation; (c) they are formed before the action; (d) they are reasons.^38^ Decisions can ground blameworthiness because they manifest individual autonomy: they are formed by deliberating moral agents and reached after the consideration and weighing of reasons. The decision to cause the harm of vision loss is the same in both A and B, which supports the view that Devin is equally deserving of blame in both these cases.^39^

Both Devin’s decision (identical in A and B) and actions (distinct in A and B) manifest, at the very least, indifference or, at worst, contempt for the interests of his victim, Angel. In both situations, Devin is committed to the achievement of his malicious goals and would, presumably, take whatever course of action was available to him. Such commitment indicates Devin’s disregard or downright disdain for his victim.

Moreover, we have good reasons to believe that Devin would choose to be in the second version of the events, which would further support his equal degree of blameworthiness for A and B. To start, Devin is to blame in both scenarios because: (i) he is a moral agent who has formed the decision to purchase chemicals in order to achieve a wrongful aim and (ii) he has taken action to carry out his decision.

By hypothesis, the decision to harm is under Devin’s control. However, the circumstances in which his decision gets carried out are not under his full control, but rather vulnerable to situational moral luck.^40^ Famously, Thomas Nagel defined moral luck as follows: “[A] significant aspect of what someone does depends on factors beyond his control, [and] yet we continue to treat him in that respect as an object of moral judgment.”^41^ The difficult question is whether the agent deserves the moral judgment (e.g., blame) given the occasion of luck.

If we take control seriously as a necessary condition for moral responsibility, then we realize that what distinguishes A from B are matters of luck, which lie outside the control of the agent whose actions are being evaluated. The luck of finding available X or XX sets into motion actions that differ in their degree of harmfulness or potential harmfulness (i.e., risks). The fact that the agent ends up imposing a 10 or 60% chance of blindness is, then, due to luck. If we ought not to hold others as blameworthy for matters that lie outside of their control, then we ought to focus on what is under their control.^42^ Although A and B present us with different luck-ridden circumstances that guide different actions, there is something over which Devin has control: his decision. This factor is identical in both cases.

Devin’s decision can ground his equal degree of blameworthiness in both A and B even though the risks that he creates are different. Furthermore, it is plausible to claim that Devin would have used XX had it been available because he would have preferred to be in scenario B, which provides additional support to the claim of equal responsibility. To see why the former claim is plausible consider the following. Devin’s deliberate choice is to pursue a harmful aim in both scenarios. Devin is a rational agent. As a rational agent, he follows the dictates of instrumental rationality, by which we mean the rationality that one employs when adopting the means that fit one’s ends. Thus, as a rational individual, Devin would pursue those means that are most likely to achieve his goal. Thus, given the opportunity, he would choose the more potent chemical XX, which means he would prefer to be in scenario B. If we divest ourselves of those elements that are not within the control of the agent (i.e., the availability of the dangerous substances), then we can see why it makes sense to state that Devin is blameworthy to the same degree in both versions of the events.^43^

The above case and like cases undermine the BTRT because the latter would offer a verdict of differential blameworthiness of agents based on the degree of risk they impose onto others. However, we have seen that a case can be made for why degrees of risk do not matter for blameworthiness in those instances when their materialization is subject to luck. In general form, the claim can be presented as follows: an agent is blameworthy if she decides to impose an unjustifiable risk of harm onto another and act on this decision. But, the degree of blameworthiness of the agent may not vary with the risk she imposes if the creation of the risk was due to luck. The BTRT holds when decisions and their implementation are free, but falters when they are subject to chance. So, once again, we have reasons to doubt the BTRT or, at least, limit its scope. Do we have reasons to believe that BTRT can survive the charge?

One set of reasons would stem from the realization that the case of Devin and Angel, as depicted above, puts forward the view that moral luck does not matter for degrees of blameworthiness.^44^ Thus, the above is reliant upon the denial of moral luck, which means that were one to accept it, then degrees of blameworthiness and risk would, once again, appear correlative.

If one accepts moral luck and, for example, denies that control is a necessary condition for responsibility, then they would arrive at different judgments vis-à-vis the degree of blame deserved by Devin. One could, then, argue that it does not matter that control (or lack thereof) played a role when either chemical X or XX was purchased: what matters is what actually happened, (i.e., differential degrees of risk were unleashed). Subsequently, the latter would ground different degrees of blameworthiness.

To tie in the differing amounts of risk to deservedness of blame, one could, then, argue that Devin is more blameworthy in B because he unleashes a greater amount of risk by relying on a preference-based view that characterizes risks as harms in themselves.^45^ On this basis the BTRT defender could then argue that Devin is more blameworthy in B because he imposes a greater risk. This is because the risk in B is more harmful than the risk in A as Angel would prefer to be exposed to the lower risk.^46^ This highlights just one possibility for recourse. There are others.^47^

It is interesting to note that the mental state of the agent problem and the reference class problem detailed before are connected. Recall that in order to rescue the BTRT from the reference class problem in the previous section, recourse was made to the decision made by the agent, John. This was a decision to impose a certain amount of subjective risk onto Mary. John’s decision grounded his blameworthiness in a way that was sensitive to the degree of risk the agent decided to impose. This section, in turn, undermined the possibility that agents’ decisions can ground differential degrees of blameworthiness that are correlative with risk under certain conditions (i.e., where the implementation of the decision is subject to situational luck). Compounded, the two problems presented dent the strength of the BTRT by limiting its scope.

## Conclusion

None of the above aims to give the impression that the BTRT is without recourse or should be abandoned. After all, the BTRT captures the significance of risks to responsibility in an intuitive and compelling way. However, the two problems illustrated in this work show that an unreserved embrace of the BTRT should be resisted.

## Footnotes

1 This assumes that, in these cases, agents are blameworthy in the first place, and that which differs is their degree of blameworthiness.

2 See, among others, Claire Finkelstein, “Is Risk a Harm?” *University of Pennsylvania Law Review*, Vol. 151, No. 3 (2003); John Oberdiek, “The Moral Significance of Risking,” *Legal Theory*, Vol. 18, No. 3 (2012); Steven Perry, “Risk, Harm, Interests, and Rights,” in Tim Lewens (ed.), *Risk: Philosophical Perspectives* (London: Routledge, 2007); Klaus Steigleder, “Climate Risks, Climate Economics, and the Foundations of Rights-based Risk Ethics,” *Journal of Human Rights*, Vol. 15, No. 2 (2016).

3 Peter F. Strawson, *Freedom and Resentment and Other Essays* (London: Routledge, 2008), pp. 1–29.

4 Ibid., p. 5.

5 It bears mentioning that it is not unusual for philosophers to speak of blameworthiness and praiseworthiness in the same breath as moral responsibility. Having said this, in this work, praiseworthiness will be more or less set to the side because the focus is on negative, as opposed to positive, outcomes and the ways in which they come about. With regard to the latter, with the exception of economics, risk is also most often conceptualized as a negative phenomenon. These considerations make blame and blameworthiness fitting center pieces of this essay.

6 Moral agency is taken to be a prerequisite for any justifiable attribution of blameworthiness (e.g., capacity to evaluate, respond, and act on reasons).

7 However, there can be other reasons why an agent ought to be blamed to a higher or lower extent. Some utilitarians, for example, might assess the utility that differing degrees of blame produced and apportion blame on these bases.

8 See Andrew von Hirsch, *Censure and Sanctions* (Oxford: Oxford University Press, 1993) and Richard Frase, “*Punishment Purposes*,” *Stanford Law Review*, Vol. 58 (2005).

9 Providing an account of degrees of blameworthiness requires the sort of treatment that goes beyond what one can achieve in the scope of one essay.

10 Although definitions of risk abound, it has become commonplace in philosophy to adopt the standard technical definition whereby risk is construed as the expectation value of unwanted events. This definition combines the probability or likelihood of a negative event with some measure of its severity or undesirability. See Stephen Luko, “Risk Management Principles and Guidelines,” *Quality Engineering*, Vol. 25, No. 3 (2013), p. 294, and Bernard L. Cohen, “Probabilistic Risk Analysis for a High-level Radioactive Waste Repository,” *Risk Analysis*, Vol. 23, No. 5 (2003).

11 We recognize that this broadly drawn divide does not do justice to the diversity of risk accounts.

12 The classic sources are Hans Reichenbach, *The Theory of Probability* (Berkeley: University of California Press, 1949) and Richard Von Mises, *Probability, Statistics and Truth* (London: Allen and Unwin, 1957).

13 The standard source is John Venn, *The Logic of Chance* (London: Macmillan, 1876). However, the appellation is found in Reichenbach, op. cit., p. 374. Also, see Alan Hájek, “The Reference Class Problem is Your Problem too,” *Synthese*, Vol. 156, No. 3 (2007).

14 A classic source is F.P. Ramsey, “Truth and Probability,” in Antony Eagle (ed.), *Philosophy of Probability: Contemporary Readings* (New York: Routledge, 2010), pp. 52-94. However, the basis for subjective interpretations of probability can be found in David Hume, *Enquiry Concerning Human Understanding* (Oxford: Oxford University Press, 2007), p. 55.

15 Bruno de Finetti, “Foresight: Its Logical Laws, Its Subjective Sources,” in Henry E. Kyburg and Howard E. Smokler (eds.), *Studies in Subjective Probability* (Malabar: Krieger Publishing Co., 1980), p. 62.

16 Andrew Ashworth, *Sentencing and Criminal Justice*. 6th ed. (Cambridge: Cambridge University Press, 2010), p. 149

17 Andrew von Hirsch and Nils Jareborg, “Gauging Criminal Harm: A Living-Standard Analysis,” *Oxford Journal of Legal Studies*, Vol. 11, No. 1 (1991), p. 1.

18 Von Hirsch, op. cit. and Frase, op. cit.

19 Venn, op. cit., p. 194.

20 Reichenbach, op. cit., p. 374

21 Reichenbach, ibid. and A.J. Ayer, “Two Notes on Probability,” in A.J. Ayer (ed.), *The Concept of a Person* (London: Palgrave, 1963), pp. 201–203.

22 Hájek, op. cit., p. 568.

23 Ibid.

24 John Oberdiek, *Imposing Risk: A Normative Framework* (Oxford: Oxford University Press, 2017), pp. 25–32, which deals with this and other difficulties related to the reference class problem.

25 Gillies, op. cit., p. 200.

26 We assume there are no reliable statistics regarding the fatality risk of car-surfing.

27 Oberdiek (2017), op. cit., p. 31.

28 It is assumed that statistical evidence exists for these reference classes.

29 Oberdiek (2017), op. cit., p. 27.

30 Gillies, op. cit., p. 53. It should be noted that there are different interpretations of Bayesian probability theories beside purely subjectivist ones. Such versions can, for example, place constraints on what counts as a rational belief. Moreover, there are other interpretations of probability (e.g., classical, logical). Gillies offers a comprehensive treatment of these and other probability theories, as well as their interconnections. Although we do not delve into these complexities, investigating such differing conceptions in relation to this paper’s topic would be fruitful. We thank an anonymous reviewer for pointing this out.

31 John Martin Fischer and Mark Ravizza, *Responsibility and Control: A Theory of Moral Responsibility* (New York: Cambridge University Press, 1998), p. 20.

32 Ishtiyaque Haji, *Deontic Morality and Control* (Cambridge: Cambridge University Press, 2002).

33 See Hájek, op. cit.

34 The act of purchasing the chemicals does not directly increase Angel’s risk of blindness. It is the adding of the chemicals to the contact lens solution that does this. However, the availability of the chemicals impacts the usage of the chemicals, which raises or lowers the risk.

35 For a defense of such a position, see Michal Zimmerman, “Taking Luck Seriously,” *The Journal of Philosophy*, Vol. 99, No. 11 (2002).

36 To be clear, desires are scalar, but it is assumed that Devin’s intensity of desire is the same in both scenarios. In any case, it is not the desires, but Devin’s decision that is taken to be the primary grounds for his deservedness of blame.

37 Ishtiyaque Haji, “Autonomy and Blameworthiness,” *Canadian Journal of Philosophy*, Vol. 24, No. 4 (1994), pp. 604-605.

38 Joseph Raz, *Practical Reason and Norms* (London: Hutchinson, 1975), pp. 488–489.

39 Some might object on the grounds that A also entails the decision to use X, while B also entails the decision to use XX. These may be decisions of the agent, but they are not free. Thus, the decisions to use X or XX do not meet the condition of decisional control.

40 Zimmerman, op. cit., p. 559. Situational luck relates to the circumstances in which the agent finds herself. If X occurs because of luck, then X is not under anyone’s control. Furthermore, control itself admits of degrees.

41 Thomas Nagel, *Mortal Questions* (New York: Cambridge University Press, 1979), p. 26.

42 Ibid., p. 25.

43 One could, of course, argue that what matters is what Devin *actually* does and not what he would do if circumstances were different. After all, we would all do different things if things were different. Are we, then, blameworthy for these things? According to Nagel, op. cit., p. 34: “We judge people for what they actually do or fail to do, not just for what they would have done if circumstances had been different.” Zimmerman, op. cit., disagrees about the normative interpretation of this statement. It is the latter’s position that is in play here.

44 In the words of Zimmerman, op. cit., p. 559: “the degree to which we are morally responsible cannot be affected by what is not in our control. Put more pithily: luck is irrelevant to moral responsibility.”

45 See Finkelstein, op. cit.

46 There are problems with such an account, and these have been sharply identified by Oberdiek (2012), op. cit., pp. 345–348.

47 For instance, on an autonomy-based view of the moral significance of risks, as described by Oberdiek (2012), op. cit., the case can be made that Angel’s autonomy, which is understood as his range of acceptable options, is curtailed to a greater extent in the second scenario because the risk to which he is exposed is higher in B. This would mean that Devin performs a more pernicious act, which in turn could spell a greater degree of blameworthiness.

